# Baicalin Induces a Potent Innate Immune Response to Inhibit Respiratory Syncytial Virus Replication *via* Regulating Viral Non-Structural 1 and Matrix RNA

**DOI:** 10.3389/fimmu.2022.907047

**Published:** 2022-06-23

**Authors:** Sheng Qin, Xianzhang Huang, Shaogang Qu

**Affiliations:** ^1^ Department of Neurology, Nanfang Hospital, Southern Medical University, Guangzhou, China; ^2^ Department of Laboratory Medicine, The Second Affiliated Hospital of Guangzhou University of Chinese Medicine, Guangzhou, China; ^3^ Guangdong-Hong Kong-Macao Greater Bay Area Center for Brain Science and Brain-Inspired Intelligence, Guangzhou, China; ^4^ Key Laboratory of Mental Health of the Ministry of Education, Southern Medical University, Guangzhou, China

**Keywords:** respiratory syncytial virus (RSV), baicalin, viral NS, viral M, L13a

## Abstract

Respiratory syncytial virus (RSV) infection is the most frequent cause of hospitalization in pediatric patients. Current systemic treatment and vaccines are not curative and re-infection is often associated with a more drastic incidence of the disease. Baicalin is a flavonoid isolated from *Scutellaria baicalensis* with potent anti-viral characteristics, namely against RSV. However, its precise mechanism of action remains unclear. Here, using *in vitro* methods and an *in vivo* murine model of RSV infection, we showed that baicalin inhibits RSV replication induces translational upregulation of type I interferons (IFNs), IFN-α and IFN-β, and reverses epithelial thickening in lung tissues. Moreover, baicalin inhibits transcription of the RSV non-structural proteins NS1 and NS2. Molecular docking and surface plasmon resonance-based affinity analysis showed that baicalin also binds to the α3 helix of the NS1 protein with an affinity constant of 1.119 × 10^−5^ M. Polysome profiling showed that baicalin inhibits translation of the RSV matrix protein (M) RNA. Baicalin mediates increased release of the ribosomal protein L13a from the large ribosomal subunit, where the extra ribosomal subunit L13a inhibits M RNA translation. These results comprehensively establish the multiple mechanisms by which baicalin induces a potent innate immune response against RSV infection.

## Introduction

Human respiratory syncytial virus (RSV) is one of the most common viruses causing infections of the lungs and respiratory tract in children worldwide. Moreover, this virus can also infect adults, specially the elderly. Despite its high incidence, to date, ribavirin remains the only available FDA approved therapy to treat RSV infection in high risk patients (1–3). RSV infection is associated with high fever, rhinitis, pharyngitis, laryngitis, bronchiolitis, and pneumonia (1). Current immunization approaches, using denatured virus particles, fail to provide long-lasting immunity against subsequent RSV infections (4–6). Furthermore, achieving an appropriate balance between immunogenicity and safety has been a great challenge in the development of a live attenuated vaccine (5). There are about 34–64 million cases of RSV-induced bronchiolitis and pneumonia in young children (<5 years old) each year worldwide, responsible for about 50,000–200,000 children (1, 7–9). RSV is an enveloped negative-strand RNA virus with a genome size of 15.2 kb that encodes 11 proteins. The RSV envelope is composed by the matrix (M), small hydrophobic (SH), fusion (F), and attachment (G) proteins (10, 11). The F and G proteins regulate the initial phases of infection (12, 13), thus being the leading targets for the development of vaccines. The M protein functions as the rate-limiting step for RSV replication within the host cell (14). RSV’s non-structural (NS) and non-homologous proteins, NS2 and NS1, function by suppressing host innate immune response, through the reduction of type I interferon (IFN) production (12, 15, 16). Type I IFN plays a crucial role in the innate immune response to viral infection (9). The mammalian type I IFNs comprise IFN-α, IFN-β, IFN-κ, IFN-δ, IFN-ϵ, IFN-τ, IFN-ω, and IFN-ζ (17–21). Of note, the molecular characterization of pathogen-associated molecular patterns (PAMPs) in RSV is still undefined (14). However, it is well documented that the expression of type I IFN is decreased or attenuated in RSV-infected cells (22). Moreover, other than suppressing type I IFN expression, NS1 and NS2 inhibit the maturation of dendritic cells, promoting, in turn, Th2 polarization and proliferation of Th17 lymphocytes (23–27). Hence, by targeting M protein, NS1 and NS2 will potentially provide a higher therapeutic index for treating RSV infection.

HEp-2 cells have been used to study RSV biology and are reported in several studies (28–30). A recent study using four different strains of RSV showed that HEp-2 cells secrete a set of different cytokines, including interleukin (IL)-6 and IL-8, along with growth factors, in response to RSV infection (31). Another study reported that RSV induces an adaptive immune response in mice *via* MyD88 and MAVS (also known as IFN-β promoter stimulator (IPS)-1) adaptors. Deletion of MyD88, in turn, showed an abnormal immune response to RSV, thus evidencing the critical role of this adaptor protein (32). IPS-1 signaling was showed to play a critical role in response to RSV infection, by mediating immune response and viral clearance (33).

Baicalin, a flavonoid isolated from *Scutellaria baicalensis*, is well known for its antiviral activity and is one of the three active components along with baicalein and wogonin, of this medical plant (34). Baicalin has been documented for its pharmacologic anti-inflammatory, anti-tumor, and anti-viral properties (34–37). Different studies, showed the potent inhibitory activity of baicalin or baicalein against RSV (38, 39). Nevertheless, the role of baicalin in RSV is not yet completely understood. Baicalin has been demonstrated to show anti-influenza A (H1N1) activity, both *in vitro* and *in vivo*, as a potent inducer of IFN-γ in CD4^+^ and CD8^+^ T cells, as well as NK cells (40). Moreover, it has been reported that baicalein increased IFN-β1 expression in RSV-infected cells, researchers have also shown that baicalin inhibits the RSV mediated production of IL-6 and -8 cytokines and regulates RSV-induced oxidative stress and signaling pathways (39). Despite the significant advances made to date, further studies are required to explore the precise mechanism of baicalin’s action against RSV. Accordingly, in this study, we aim to uncover baicalin mechanism of action in a context of RSV infection. Our findings showed that baicalin inhibited the translation of viral M protein and transcription of the viral NS2 and NS1 proteins. Additionally, baicalin was showed to inhibith the expression of other inflammatory cytokines, while inducing expression of type I IFNs, thus mounting a potent anti-RSV innate immune response.

## Materials and Methods

### Ethics

All animal experiments were performed according to protocols validated by the Institutional Animal Care and Use Committee of the First Affiliated Hospital of Guangzhou Medical University (Approval number: 2018-25). The studies were performed in accordance with the principles of Declaration of Helsinki and observed the Interdisciplinary Principles and Guidelines for the Use of Animals in Research, Testing, and Education by the New York Academy of Sciences, *Ad Hoc* Animal Research Committee.

### Cell Culture and Treatment

The lung adenocarcinoma HEp-2 cell line, acquired from American Type Culture Collection (ATCC, USA), was cultured in Dulbecco’s Modified Eagle Medium (DMEM, Invitrogen, USA)supplemented with 10% fetal bovine serum (FBS) and 1% penicillin streptomycin (Invitrogen, Carlsbad, CA, USA). All cell lines were kept at 37°C in 5% CO_2_. Cells were treated with different doses of baicalin resuspended in DMEM (Shanghai Aladdin Biochemical Technology Co., Ltd, China) for 72 h.

### Viral Growth, Infection, and Plaque Inhibition Assays

RSV A2 strain was acquired from ATCC and grown as previously described (13). Briefly, titer was ascertained using HEp-2 cell monolayers and further purified using sucrose density gradients. Samples were aliquoted and stored at −80°C until further utilization. The infection of RSV was done using a multiplicity of infection (MOI) of three. Furthermore, 4 h post-infection with RSV, the medium was changed to remove any nonadsorbed RSV particles. For the plaque inhibition assay, HEp-2 cells (1 million cells/well) were seeded onto 6-well plates. An equal volume of RSV suspension (MOI=3) was used and subsequently, the plates were then incubated for 1 h at 36°C with shaking. After 1 h, 3 ml of 0.6% agarose supplemented with 4% FBS, and the indicated doses of baicalin was added to each well. Subsequently, the plates were incubated at 36°C, 5% CO_2_ for 72 h. Cells were stained with crystal violet (0.1%) overnight, imaged using a 20x magnification, and the number of infected cell foci were counted in 20 different areas and averaged. Percent reduction was calculated as inhibition relative to the infected cells that were not processed with baicalin.

### Cytotoxicity Assay

Cells were processed with enhancing doses of baicalin in 96-well format (3,000 cells/well). Cell viability after 72 h was measured using the CCK-8 assay (Sigma-Aldrich, St. Louis, USA). Absorbance at 570 nm was plotted relative to mock-treated controls, and the half toxic concentration (TC_50_) was ascertained.

### Immunofluorescence Staining

RSV immunofluorescence staining was performed as previously described (41) and using an anti-RSV antibody (Diagnostic Hybrids, OH, USA). Images were obtained through confocal laser scanning microscopy (Zeiss LSM 510 Meta, Carl Zeiss MicroImaging, USA).

### Determination of *In Vivo* Effect of Baicalin

Six-week-old male BALB/c mice (18-20 g) were obtained from the Laboratory Animal Center of Guangzhou University of Chinese Medicine and housed in pathogen-free conditions with unrestricted access to water and food. Mice were anesthetized by ether narcosis and infected intranasally with 5 × 10^6^ PFU of RSV (n = 20). 24 h after infection, mice were randomly categorized into four groups (n = 5/group), and treaded as follow:

Group 1: No treatment
Group 2: Oral administration of 100 mg/kg baicalin
Group 3: Oral administration of 200 mg/kg baicalin
Group 4: Oral administration of 50 mg/kg ribavirin

The treatments were administered daily, for five consecutive days. Ten uninfected mice were also randomly divided into two groups (n = 5/group) and treated as follow:

Group 1: No treatment
Group 2: Oral administration of 200 mg/kg baicalin

The weight of mice was assessed on each day of the experiment. Blood was collected by orbital bleeding on day 5 and processed for enzyme-linked immunosorbent assay (ELISA) as described below. Mice were euthanized on day 5, and lung tissues were processed for hematoxylin and eosin (H&E) staining using standard methodologies. Homogenates were used for polysome profiling and determination of viral RNA content through RSV real-time quantitative polymerase chain reaction (RT-qPCR) kit (Liferiver, La Jolla, CA, USA).

### Polysome Profiling

Polysome profiling was performed as previously described (14). Lung homogenates were processed in lysis buffer: Triton X-100, 100 mM KCl, 1% (v/v), 5 mM MgCl_2_, 10 mM Tris-Cl, pH 7.4, 1000 U/ml RNasin, 2 mM DTT, 0.5% (w/v) deoxycholate, and 100 µg/ml cycloheximide. Post-nuclear extracts were layered on 10–50% sucrose gradients, followed by ultracentrifugation at 100,000 × g using a SW41 rotor (Beckman, Kraemer Boulevard Brea, CA, USA), for 4 h. Gradients were fractionated using a BR-184 tube piercer (Brandel, Gaithersburg, MD, USA) and a UA-6 UV detector (Teledyne ISCO, USA). Digital outcomes were collected with the help of a DI-158U USB (DATAQ Instruments, USA) and monitored at 254 nm over time with the Peak Chart Data Acquisition Computer Program.

### RNA Isolation and RT-qPCR

Total RNA from cells or polysome fractions were extracted with TRIzol (Thermo Fisher Scientific, Waltham, MA, USA) according to manufacturer instructions. Synthesis of cDNA was performed using SuperScript III reverse Transcriptase (Thermo Fisher Scientific, Waltham, MA, USA), and the RT-qPCR reactions conducted using KAPA SYBR FAST (KAPA BIOSYSTEMS, Boston, MA, USA). The PCR primers used are listed in **Table 1**. Raw Ct values were normalized to the *Actb* gene, and fold changes were evaluated by employing the 2^-ΔΔCt^ approach.

**Table 1:** Oligonucleotides used for RT-qPCR.

Gene name	Forward primer (5′ - 3′)	Reverse primer (5′ - 3′)
*NS1*	GAATGGCATTGTGTTTGTGC	TGGCATTGTTGTGAAATTGG
*NS2*	TGCACAAAGTGGGAAGCAC	TGCCAATGCATTCTAAGAACC
*N*	TGCAGGGCAAGTGATGTTAC	TTCCATTTCTGCTTGCACAC
*P*	GGCAAGACTCAGGAATGAGG	TCCCTTCCAACAGGTTGTTC
*M*	AATGCCCAGCAAATTTACCA	GCCTTGATTTCACAGGGTGT
*SH*	CCAATCTGATGGCACAAAAC	GCTTGCATGGTGAGATGTTG
*G*	AAGTCAACCCTGCAATCCAC	TTTGTTTTGGCGTTGTTTTG
*F*	TAGGAGCCATTGTGTCATGC	ATCGCACCCGTTAGAAAATG
*M2*	CCCATGCACTGCTTGTAAGA	CCAACTCTGCAGCTCCACTT
*Actb* human	CACCATTGGCAATGAGCGGTTC	AGGTCTTTGCGGATGTCCACGT
*Actb* mouse	CATTGCTGACAGGATGCAGAAGG	TGCTGGAAGGTGGACAGTGAGG
*Ifna1*	GGATGTGACCTTCCTCAGACTC	ACCTTCTCCTGCGGGAATCCAA
*Ifnb1*	GCCTTTGCCATCCAAGAGATGC	ACACTGTCTGCTGGTGGAGTTC
*Tnf*	GGTGCCTATGTCTCAGCCTCTT	GCCATAGAACTGATGAGAGGGAG
*I110*	CGGGAAGACAATAACTGCACCC	CGGTTAGCAGTATGTTGTCCAGC
*I16*	TACCACTTCACAAGTCGGAGGC	CTGCAAGTGCATCATCGTTGTTC

### Quantification of Cytokines

IFN-α, IFN-β, TNF-α, IL-10, and IL-6 were in plasma samples obtained from mice infected with RSV, using ELISA kits specific for each cytokine (Abcam, Cambridge, MA, USA).

### Western Blot Analysis

Cells were washed with ice-cold PBS and lysed with radioimmunoprecipitation (RIPA) lysis buffer (Thermo Fisher Scientific, 89900) at the end of the experimental time point. Before proteins were run on SDS-PAGE gels and transferred to PVDF membranes, concentrations were ascertained using a BCA protein assessment kit (Thermo Fisher Scientific, Waltham, MA, USA). Antibodies including anti-L13a (ab96074) and anti-L11 (ab79352) used to probe the blots were acquired from Abcam (Cambridge, MA, USA). Anti-phospho L13a antibody was generated as previously described (42). Anti-NS1, anti-NS2, and anti-M antibodies were produced by AtaGenix (Wuhan, China). To confirm the equal protein loading for all the conditions tested, blots were immunoblotted using β-Actin antibody (ab8226) purchased from Abcam(Cambridge, MA, USA).

### Molecular Docking

#### Receptor Structure Preparation

The amino acid chain of the PDB ID: 5VJ2 protein structure was used as the receptor for molecular docking. By taking advantage of AutoDockTools 1.5.6 (43), the primary structure was processed to preserve the original charge of the protein and to generate pdbqt files for docking.

#### Ligand Structure Preparation

The docked ligand baicalin was downloaded from the PubChem website (ID: 64982). At the experimental condition of pH = 7.4, the carboxyl group in the baicalin structure was deprotonated. The Molecular Orbital PACkage (MOPAC) program (44) was used to optimize the molecular structure and calculate the atomic charge of Austin Model I (AMI) (45), and the ligand structure was then processed using autodock tools 1.5.6. After processing, pdbqt files were created for docking.

#### Molecular Docking Method

Molecular docking was performed using the AutoDock 4.2.6 program (46). First, based on the data available in the literature, it was determined that the ligand docking site is near the α3 helix of the NS1 protein, and the center point coordinates of the docking interface bag were set to (8.45, 16.46, and 77.12), 40 × 50 × 40 was the grid points in each direction of the docking box XYZ. The spacing was set to 0.375 Å, the docking time was 100, and the other parameters applied were default values. All calculations were done on the MolDesigner Molecular Simulation Platform.

### Surface Plasmon Resonance (SPR)

The affinity of NS1protein with baicalin was determined by SPR using Biacore T200. The CM5 chip was coupled to NS1, and baicalin with different indicated concentration gradients were allowed to flow across the surface of the chip. The affinity constant (KD) was determined from the ratio of the binding constant (ka) and dissociation constant (kd).

### Statistical Analysis

Results presented in this manuscript, unless otherwise mentioned, are representative of three independent experiments, and correspond to the mean ± standard deviation (SD). Intergroup explorations were exerted by implementing the one-way analysis of variance (ANOVA) succeeded by Tukey’s *post hoc* test or unpaired non-parametric Wilcoxon-Mann-Whitney (WMW) test for several comparisons. A *P* < 0.05 was considered statistically significant.

## Results

### Baicalin Exhibits Anti-RSV Activity Both *In Vitro* and *In Vivo*

**Figure 1:**
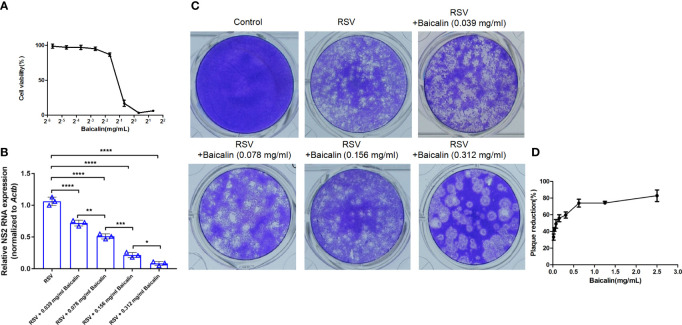
Baicalin demonstrates potent anti-RSV activity *in vitro*. **(A)** The cytotoxic influence of baicalin was determined by assessing cell viability by CCK-8 assay. HEp-2 cells were treated in triplicate with baicalin increased concentrations for 72 h. Absorbance at 570 nm in relation to that of mock treated control was plotted for evaluation of the TC_50_ value. **(B)** Expression of viral transcripts of the NS2 gene was analyzed by RT-qPCR in HEp-2 cells infected with RSV (MOI = 3) and treated with the indicated doses of baicalin. Data were normalized to *Actb* expression. Results are shown as mean ± SD (n = 3); *P < 0.05, **P < 0.01, ***P < 0.001, ****P < 0.0001 – ANOVA with Tukey’s post-hoc test. **(C, D)** Baicalin IC_50_ was determined through plaque reduction assessment in HEp-2 cells infected with RSV in the presence of the indicated doses of baicalin **(C)**. The number of plaques was counted and plotted as the percentage of plaque inhibition in relation to untreated cells, after 72 h **(D)**.

*In vitro* treatment with baicalin revealed a TC_50_ of 0.449 mg/ml (**Figure 1A**). Treatment with baicalin significantly reduced the immunofluorescent signal for cytosolic RSV N protein in a dose-dependent manner (**Supplementary Figures 1A–E**), demonstrating that baicalin effectively inhibits RSV infection by attenuating viral replication. To examine the inhibitory effects of baicalin on RSV replication, HEp-2 cells were infected with RSV (MOI = 3) and treated with increasing concentration of baicalin for 72 h. The synthesis of viral NS2 RNA was assessed by RT-qPCR. Baicalin treatment inhibited the synthesis of NS2 RNA in a dose-dependent manner (**Figure 1B**). A plaque assay performed in HEp-2 cells revealed that the concentration that inhibited 50% of the virus (IC_50_) for RSV (MOI = 3) *in vitro* was 0.0894 mg/ml baicalin after 72 h (**Figures 1C, D**). Virus reduction by nonspecific cytotoxicity was ruled out, as even the highest dose of baicalin used for anti-RSV activity, did not induce significant cell cytotoxicity. Taken together, these outcomes elucidated the fact that baicalin hindered RSV replication *in vitro*.

**Figure 2:**
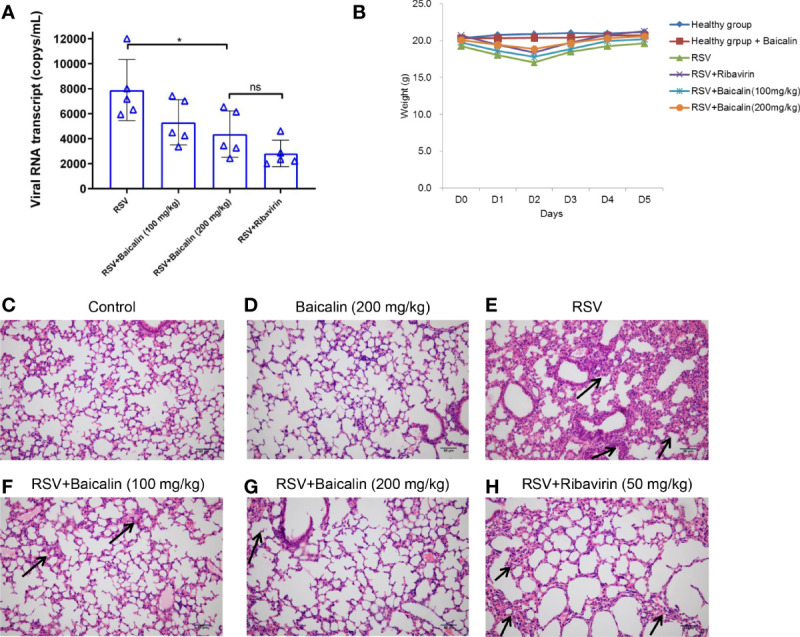
Baicalin exhibits potent anti-RSV activity *in vivo*. **(A)** Expression of viral transcripts of RSV RNA was evaluated by RT-qPCR in lung homogenates obtained from RSV-infected mice, either untreated or treated with ribavirin or baicalin. Data is shown as mean ± SD (n = 5); *P < 0.05, *ns*, not significant – ANOVA with Tukey’s *post-hoc* test. **(B)** Weight of mice over five days in the different experimental groups.No significant difference in weight was observed for any of the experimental groups. **(C-H)** Male BALB/c mice were infected with RSV on the first day and were either left untreated **(E)** or treated with ribavirin **(H)** or increased concentrations of baicalin for five days **(F, G)**. The presence of hyperplasia is indicated by the arrows. Healthy mice untreated **(C)** or treated with baicalin **(D)** were euthanized on day 5 and processed for H&E staining. Epithelial cell thickening observed in RSV-infected mice was ameliorated when treated with baicalin. Scale bar = 80 µm.

*In vivo*, BALB/c mice were infected with RSV intranasally and treated with increasing concentrations of baicalin or ribavirin once a day for five days. The inhibitory effect on virus growth was indirectly evaluated by detecting the transcription levels of RSV mRNA by RT-qPCR. Baicalin treatment resulted in significant inhibition of viral RNA transcript compared to untreated conditions (**Figure 2A**). Inhibition of viral replication with high doses of baicalin was not significantly different compared to ribavirin treatment (**Figure 2A**). At day 5, no differences in body weight were observed between uninfected or infected mice ± baicalin or ribavirin (**Figure 2B**). However, as indicated by the arrows, H&E staining revealed epithelial cell thickening or hyperplasia in mice infected with RSV compared to healthy mice ± baicalin (**Figures 2C–E**). Hyperplasia occurs due to epithelial cell injury and the subsequent proliferation of nearby epithelia to replace the injured cells. Treatment with baicalin showed a dose-dependent improvement in lung hyperplasia (**Figures 2F, G**). In comparison, ribavirin-treated RSV-infected mice had comparatively more evidence of hyperplasia (**Figure 2H**). Taken together, these results provide evidence that baicalin has potent anti-RSV activity both *in vitro* and *in vivo*.

### Baicalin Promotes Translational Activation of Type I Interferon and Suppresses Pro-Inflammatory Cytokines

**Figure 3:**
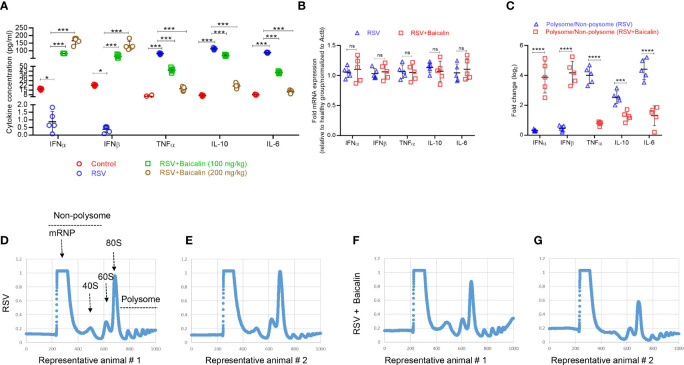
Baicalin promotes type I interferon (IFN) activation. **(A)** Plasma levels of type I IFNs (IFN-α and IFN-β) and pro-inflammatory cytokines (TNF-α, IL-10, and IL-6) in RSV-infected mice ± baicalin (after five days) were determined by ELISA. **(B)** Expression of transcripts encoding IFN-α, IFN-β, TNF-α, IL-10, and IL-6 in lung homogenates recovered from RSV-infected mice, either untreated or treated with baicalin. Data was normalized to *Actb* expression. **(C)** Polysomal association of transcripts encoding IFN-α, IFN-β, TNF-α, IL-10, and IL-6 in lung homogenates obtained from RSV-infected mice, either untreated or treated with baicalin. Ribosomal fractions isolated from RSV-infected mice ± baicalin were exposed to 10–50% sucrose gradient centrifugation. The purification of total RNA from each fraction was carried out and then used for RT-qPCR with the gene-specific primers listed in **Table 1**. The ratio of polysomal to non-polysomal mRNA amount was evaluated and plotted for each gene. Data shown in **A-C** represents mean ± SD (n = 5); *P < 0.05, ***P < 0.001, ****P < 0.0001, *ns*, not significant – ANOVA with Tukey’s *post-hoc* test. **(D–G)** Representative polysome profiles obtained from lung homogenates of RSV-infected mice, untreated **(D, E)** or treated with baicalin **(F, G)**. Similar polysome profiles showed that treatment with baicalin did not result in global translation inhibition but instead in transcript-specific inhibition as observed in **C**.

Blood was collected by orbital bleeding from mice infected with RSV and either untreated or treated with baicalin. In untreated RSV-infected mice, the expression of type I IFNs, IFN-α and IFN-β, was lower compared to the basal expression observed in healthy mice. Baicalin treatment favors a dose-dependent increase in type I IFNs, IFN-α, and IFN-β in RSV-infected mice (**Figure 3A**), indicating that baicalin treatment results in the activation of type I IFNs in these mice. The pro-inflammatory cytokines TNF-α, interleukin (IL)-10, and IL-6, which are associated with the development of an innate immune response, were increased in RSV-infected mice. Baicalin treatment suppressed the levels of the pro-inflammatory cytokines (**Figure 3A**). These findings suggest that baicalin suppresses proinflammatory cytokines.

To determine whether the increase in plasma levels of type I IFNs and pro-inflammatory cytokines occurred at the transcription level, RT-qPCR was used to identify the comparative level of mRNA in lung homogenates of the different experimental groups. There was no considerable discrepancy in mRNA expression of *IFN-α*, *IFN-β*, *TNF-α*. *IL-10*, and *IL-6* in RSV-infected mice ± baicalin (200 mg/kg) (**Figure 3B**). This indicates that baicalin-induces an increase in IFN-α and IFN-β levels, and a decreases on TNF-α, IL-10, and IL-6 at the post-transcriptional level.

We next performed polysome profiling of the lung homogenates. RT-qPCR analysis was done on RNA isolated from the non-translating, non-polysome, and actively-translating polysome pools. The ratio of mRNA in polysome and non-polysome pools, indicative of their translational index, was plotted. Baicalin induced increased polysome occupancy of IFN-α and IFN-β, and decreased polysome occupancy of TNF-α, IL-10, and IL-6 (**Figure 3C**), indicating that baicalin regulates the translation of these genes. The change in translation was transcript-specific and not global, as demonstrated by the similar polysome profiles observed in RSV-infected animals untreated or treated with baicalin (**Figures 3D–G**).

### Baicalin Binds NS1 and Regulates Transcription and Translation of Different RSV Genomic Components

**Figure 4:**
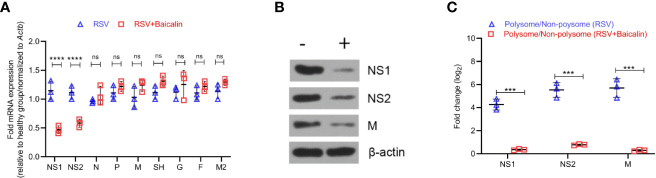
Baicalin affects transcriptional and translational regulation of RSV gene products. **(A)** Baicalin causes the downregulation of viral genes NS1 and NS2, but not others. Expression of indicated viral transcripts in lung homogenates of RSV-infected mice ± baicalin was quantified by RT-qPCR. Outcomes were normalized to *Actb*. **(B)** Immunoblot analysis of NS1, NS2, and M protein in lung homogenates of RSV-infected mice ± baicalin showed downregulation in the expression of these proteins after treatment with baicalin. Representative blots from 3 animals are shown. β-actin was immunobloted as a loading control. **(C)** Polysome occupancy of NS1, NS2, and M RNAs. Baicalin caused translational downregulation of viral M RNA. The decrease in polysome occupancy observed for NS1 and NS2 is attributable to the decrease in their total RNA. Data shown in **(A**, **C)** represents mean ± SD (n = 5); ***P < 0.001, ****P < 0.0001, *ns*, not significant – ANOVA with Tukey’s *post-hoc* test.

Given our observed decrease in viral replication, after the treatment with baicalin *in vito* and *in vivo*, we next determined the effect of baicalin on RSV genome RNAs. Baicalin treatment significantly reduced transcription of NS1 and NS2 mRNA (**Figure 4A**). Immunoblot analysis confirmed a decrease in NS1 and NS2 protein in lung homogenates of baicalin-treated mice compared to untreated RSV-infected mice (**Figure 4B**). In addition, the viral protein M, which is the rate-limiting factor for RSV replication, was also downregulated after baicalin treatment (**Figure 4B**). However, no significant changes in M mRNA levels were observed (**Figure 4A**). We next analyzed the polysome fractions for other viral RNAs. The polysome occupancies of NS1 and NS2 RNA were significantly downregulated after baicalin treatment (**Figure 4C**), which is in agreement with the decreased transcript levels observed (**Figure 4A**). Polysome occupancy of viral M RNA was also significantly downregulated in the post-baicalin treatment group (**Figure 4C**), indicating that baicalin inhibits translation of viral M RNA.

The effect of baicalin on RSV replication can either be mediated by direct binding to one of the viral proteins or by an indirect secondary mechanism. The NS1 protein structure contains three α helices and seven β sheets, from which the C-terminus α3 helix was showed to have a critical role in innate immune regulation (24). The NS1 protein is a dimer in the crystal structure of 5VJ2. Chatterjee and coworkers used multi-angle light scattering (Size exclusion chromatography-multi-angle light scattering; SEC-MALS) and size exclusion chromatography to further confirm that NS1 is a dimer. Under these conditions, it is 17.5 ± 2.1 kDa, indicating that the monomer may be the smallest unit for its physiological function (24). Based on this, we hypothesize that the baicalin molecule is likely to bound near the α3 helix of a single NS1 protein, thereby affecting its function. The results of molecular docking simulations indicated that baicalin can bind to the α3 helix on the NS1 protein, with a binding energy of -5.01 kcal/mol (**Supplementary Figure 2A**). The docking results indicate that baicalin was mainly bound to the grooves on the surface of NS1 protein and to the hydrophobic surface of NS1 protein through hydrophobic interactions.

**Figure 5:**
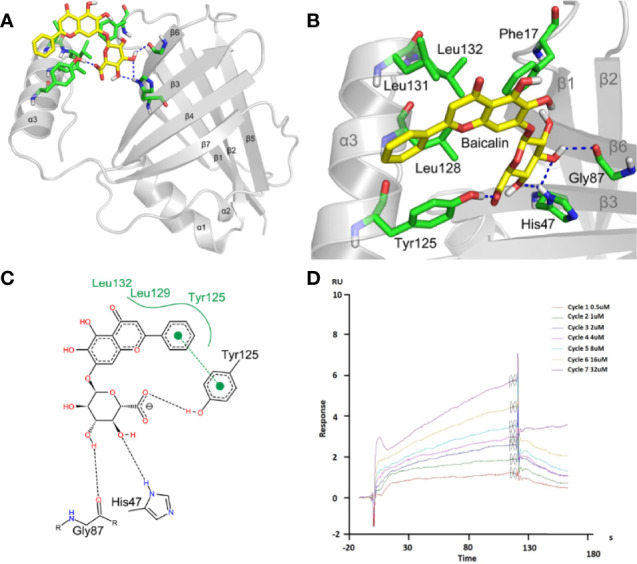
Baicalin binds to viral NS1 protein with high affinity. **(A-C)** Analysis of the interaction between baicalin and NS1 protein. The yellow stick indicates baicalin, the green stick indicates the interacting amino acids, and the blue dashed line indicates hydrogen bonding **(A, B)**. The black dotted line represents the hydrogen bonding, the green solid line the hydrophobic interaction, and the π-π stacking is represented by solid circles and dashed lines **(C)**. **(D)** Surface plasmon resonance (SPR) analysis of baicalin binding to viral NS1 protein. NS1 was coupled to the CM5 chip. Representative response curve of NS1 and baicalin binding is shown.

To further study the mechanism for the recognition of baicalin and NS1 protein, we conducted an in-depth exploration of their binding dynamic (**Figure 5A**). Baicalin bonded to the pocket between the α3 helix, β3, and β6, and was predicted to form strong hydrogen bond interactions with the surrounding amino acid residues (**Figures 5B, C**). The baicalin molecules were mainly bound by Phe17 (phenylalanine), His47 (histidine), Gly87 (glycine), Tyr125 (tyrosine), Leu128 (leucine), Leu131, and Leu132 amino acid residues in the pocket, among which the polyhydroxy part formed hydrogen bonds with His47, Gly87, and Tyr125 of the NS1 protein. The benzene ring part formed a strong hydrophobic interaction with Tyr125, Leu129, and Leu132, and formed π-π stacking interactions with the benzene ring of Tyr125 (**Figures 5B, C**). According to the binding mode of baicalin and NS1 protein, we found that hydrogen bonding, π-π stacking, and hydrophobic interactions are important driving forces for the combination of the two. To confirm the predicted physical interaction of baicalin and NS1, we performed SPR-based affinity analysis. The response curve revealed a dynamic interaction of NS1 and baicalin (**Figure 5D**), with an affinity constant (KD) of 1.119 × 10^−5^ M (**Supplementary Figure 2B**).

### Baicalin Promotes the Release of Ribosomal Protein L13a That Inhibits Translation of Viral M RNA

**Figure 6:**
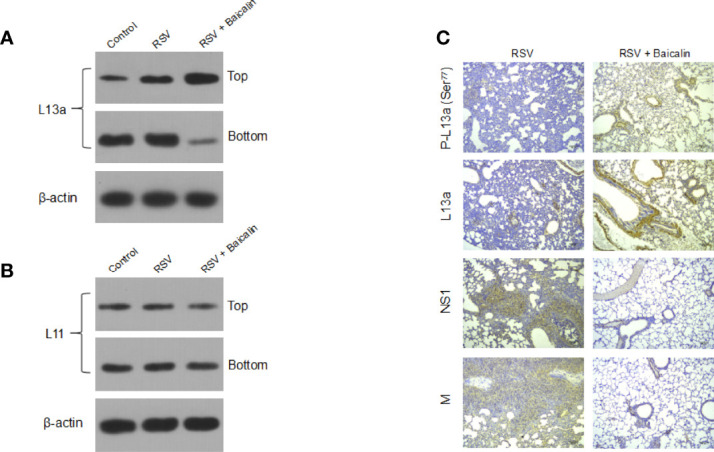
Baicalin represses the translation of RSV M RNA by inducing the extra ribosomal release of the cognate repressor protein L13a. **(A, B)** Baicalin induced an increase in the release of L13a from ribosomes in mice RSV-infected lungs, as demonstrated by immunoblotting **(A)**. This release was specific for L13a, as no effect was seen on another larger subunit protein L11 **(B)**. *Top* and *bottom* refer to the released proteins and ribosome sediments, respectively, collected post-sucrose gradient centrifugation using the lung homogenates. Representative blots for one mouse in each group (n = 3) are shown. **(C)** Immunohistochemistry analysis of L13a, phosphorylated L13a, and RSV viral NS1 and M proteins in lung tissues of RSV-infected mice ± baicalin. Scale bar = 80 µm.

It was previously shown that the viral M RNA has a *cis*-acting element in its 3′-untranslated region (3′-UTR), termed the respiratory syncytial virus-activated inhibitor of translation (VAIT) (14). After RSV infection, the ribosomal protein L13a was shown to be released from the 60S ribosomal subunit (14). The extra ribosomal L13a protein was shown to bind the VAIT element in the M RNA and inhibit its translation (14). Hence, we determined whether baicalin-mediated translation inhibition of M RNA was being mediated by the extra ribosomal L13a. Lung homogenates from RSV-infected mice, untreated or treated with baicalin, were subjected to centrifugation over a 20% sucrose cushion as described previously (14), which separated the released proteins to the lighter fraction while secluding the ribosomal aggregates to the bottom of the cushion. Immunoblot analysis of the top and bottom fractions showed that RSV infection, by itself, resulted in the release of L13a from the ribosomes (**Figure 6A**) compared to uninfected mice. The treatment of baicalin led to a robust enhancement in the release of L13a (**Figure 6A**). This was specific for L13a, as no extraribosomal release of another ribosomal protein L11 was observed (**Figure 6B**). Indeed, immunohistochemistry revealed increased expression of both L13a and P-L13a proteins, and decreased expression of viral NS1 and M proteins, in the lungs of RSV-infected mice treated with baicalin (**Figure 6C**). These results indicate that baicalin potentiates innate immune response against RSV infection at multiple levels. This may be due either by the transcriptionally downregulation of NS1 and NS2, physical interaction with NS1 protein, or induction of the L13a release, which in turn mediates translation inhibition of the rate-limiting factor for RSV replication, the M protein.

## Discussion

Potent anti-viral effects of baicalin have been previously demonstrated (37–39). However, baicalin-mediated inhibition of RSV, as well as the cellular mechanism behind it remain poorly understood. In this study, we explored the action mechanism of how baicalin induces anti-RSV activity at multiple levels. To date, there is no report on how baicalin regulates RSV, NS1, and NS2 transcripts and proteins. Accordingly to this work, we demonstrated that baicalin significantly inhibited viral replication, as assessed by RSV transcript quantification. In addition, we showed that baicalin regulates NS1 and NS2 mRNA levels in a dose-dependent manner. Although a previous study from Groves et al. (47) showed that RSV infection induced weight loss by changes in gut microbiota, in our studies, we did not find any difference in the bodyweight between infected mice versus the control group. RSV has been shown to infect epithelial cells and is reported to increase the permeability of the airway epithelial barrier and change the molecular composition of epithelial junctions by regulating its protein expression (48). Duan et al. demonstrated the role of baicalin on the attenuation of LPS induced alveolar epithelial A549 cells injury, *via* FSTL1 signaling pathway, and more specifically by upregulating miR-200b-3p expression (49). Another study conducted by Yoshida et al. showed that baicalin was able to suppress type 2 immunity by intersecting the interplay between mast cell and airway epithelial cells (50).In our study, we report that RSV infection induces epithelial cells injury and affects their ability to proliferate and replace the injured cells. Treatment with baicalin showed a dose-dependent improvement in lung hyperplasia.

Researchers have discovered that the type I IFN response (IFN-α/IFN-β) exhibits a pivotal role in the inhibition of influenza infection (51). The role of type I IFN in the response to RSV infection is critical in modulating the viral clearance rate and in guiding the immune response (12). It has been shown that baicalin inhibited influenza virus A replication *via* activation of IFN related signaling by reducing miR-146a (52) and resulted in the reduction of pro-inflammatory cytokines (38). Another study has reported that baicalin possesses anti-influenza virus activity and acts as a potent inducer of IFN-γ production (40). Lin et al. showed that baicalin reduced RSV induced oxidative stress, malondialdehyde production, and inhibited NF-κB, COX-2, Stat3, and MAPK (39). Consistent with the above findings, our study found that baicalin promots the activation of type I IFNs and inhibits pro-inflammatory cytokines. We also validated the baicalin-mediated effect on IFN-α and IFN-β, and found that they are upregulated, thus indicating that baicalin plays a crucial role in regulating the polysome formation of specific transcripts rather than global transcripts.

The RSV NS1 and NS2 proteins have been demonstrated to antagonize IFN-mediated host responses through regulating both type I and III IFN induction (16, 53). NS1 interacts with MAVS to suppress phosphorylation of IRF-3 (54). Additionally, NS proteins have been reported to participate in “NS-degradosome” formation that facilitates the degradation of IFN induction or signaling components, such as RIG-I, TBK1, IRF-7, IRF-3, and STAT2 (55). Different studies have also shown that NS1 suppresses the proliferation and activation of the protective cell populations (CD103^+^ CD8^+^ T cells and Th17 cells) while facilitating the proliferation and activation of Th2 cells that enhance the RSV infection. Since the role of baicalin has not been defined, in this study, we found an association of baicalin with both of these nonstructural proteins, and hereby report that baicalin treatment significantly decreased the expression of NS1 and NS2 transcript and protein expression, which reflects its protective effects (26). Next, we analyzed how baicalin is associated with NS1 and molecular docking results showed that baicalin binds to the α3 helix of NS1 with a bind energy of -5.01 kcal/mol. Further, in-depth analysis revealed that baicalin is likely to bind the pocket between α3 helix, β3, and β6 to form a strong hydrogen bonding. The benzene ring is involved in a hydrophobic interaction with Tyr125, Leu129, and Leu132, and formed π-π stacking interactions, highlighting a crucial role of baicalin in regulating NS1 protein. Taken together, these results suggest that baicalin presents a great antiviral activity against RSV, by inhibiting some of the virus key components.

Baicalin has been demonstrated to have anti-viral activity against different RNA viruses (56–58). In all of these cases, whether the mechanism of antiviral activity is similar, remains to be determined. In the case of influenza virus, it has been shown that baicalin inhibits NS1 interaction with the p-85β subunit *via* Src homology 3 (SH3) binding motif, in turn inhibiting the PI3K-Akt signaling pathway (59). Whether NS1 interacts with the p-85β subunit in RSV-infected host cells or baicalin inhibits that interaction remains to be elucidated.

The M protein is a non-glycosylated phosphorylated protein located in the outer part of the nucleocapsid layer, which serves as a bridge between the nucleocapsid and the lipid bilayer envelope (60, 61). The M protein drives the coordination between viral structural components and accelerates virus assembly by connecting the nucleocapsid and lipid bilayer envelope (60, 62). The M protein is in charge of reconstituting other synthetic viral proteins into a new and complete virus (63). Therefore, in the absence of the M protein, recombination would not take place RSV virus will not replicate (63, 64). Our work revealed that the effect of baicalin on the inhibiting translation of the M RNA—which was the limiting factor for RSV replication—was mediated by the release of L13a from the large ribosome. L13a is known to regulate the translation of host pro-inflammatory cytokines by binding to a *cis*-acting element called GAIT element (14, 42, 65). Guan et al. demonstrated the role of ribosomal protein L13 (RPL13) in regulation of antiviral immune response induced by foot-and-mouth disease virus (FMDV) (66).However, it remains uncertain how RSV infection induces L13a dissociation and release, and the strategy by which baicalin further potentiates this.

GAIT-element mediated regulation of IFNγ activates death-associated protein kinase-1 (DAPK1) which phosphorylates and activates zipper-interacting protein kinase (ZIPK), that will in turn mediate L13a phosphorylation (67) and induce its release from the large ribosomal subunit (52). In our study, we observed an increased expression of both L13a and phosphorylated L13a in lung tissues of RSV-infected mice treated with baicalin. Whether baicalin activates DAPK1/ZIPK mediated phosphorylation and release of L13a, or whether a separate mechanism is involved, remains to be determined.

**Figure 7:**
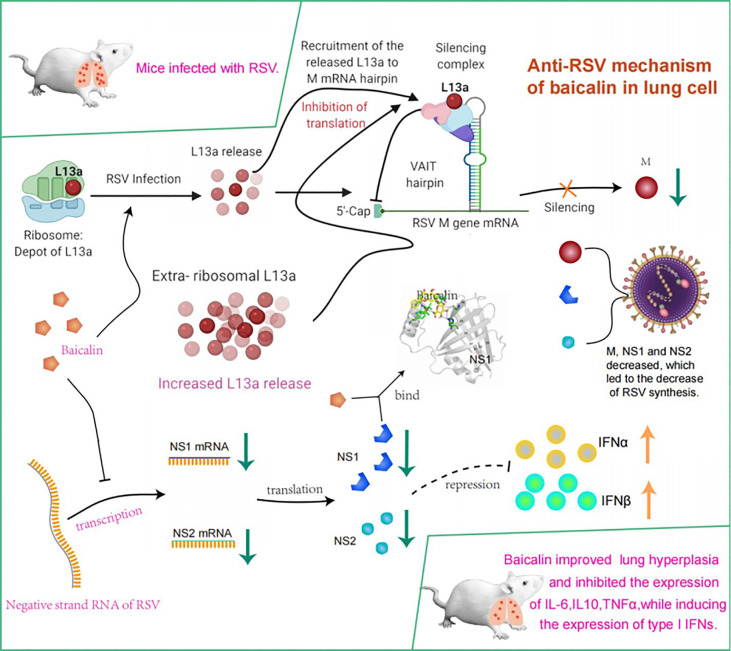
Model summarizing mechanism underlying potent innate immune response elicited against RSV infection by treatment with baicalin. Translational regulation of RSV viral M RNA was previously elucidated (14). Briefly, baicalin suppresses translation of the viral M protein and transcription of the viral NS1 and NS2 proteins and downregulates the expression of pro-inflammatory cytokines while inducing expression of type I IFNs, thus mounting a potent anti-RSV innate immune response.

In conclusion, we confirmed that baicalin inhibited translation of the viral M protein and transcription of the viral NS1 and NS2 proteins. We also showed that baicalin inhibited the expression of pro-inflammatory cytokines, while inducing the expression of type I IFNs, ultimately mounting a potent anti-RSV innate immune response (**Figure 7**). This discovery reported an in-depth view of baicalin’s role and importance in a context of RSV infection. Baicalin as thus a great potential as therapeutic candidate for the treatment of RSV. However, our data regarding the regulatory effect of baicalin on RSV remain limited, since we only investigated the impacts of baicalin on viral M protein, NS1 and NS2. Further studies are thus crucial to determine the regulatory effect of baicalin on other aspects for RSV treatment.

## Data Availability Statement

The original contributions presented in the study are included in the article/**Supplementary Material**. Further inquiries can be directed to the corresponding authors.

## Ethics Statement

The animal study was reviewed and approved by the First Affiliated Hospital of Guangzhou Medical University (Approval number: 2018-25).

## Author Contributions

SGQ is the senior and corresponding author. SGQ and SQ conceived and designed the study. SGQ supervised the study. SQ performed experiments, collected the data, and prepared the manuscript. SGQ and XH provided critical materials and revised the manuscript. All authors have read and approved the final version of the manuscript.

## Funding

This work was supported by grants from the Science and Technology Planning Project of Guangdong Province (2021A1515011507) and Chinese Medicine Science and Technology Research Project of Guangdong Provincial Hospital of Chinese Medicine (YN2019QL05). The funders had no role in study design, data collection and analysis, decision to publish, or preparation of the manuscript.

## Conflict of Interest

The authors declare that the research was conducted in the absence of any commercial or financial relationships that could be construed as a potential conflict of interest.

## Publisher’s Note

All claims expressed in this article are solely those of the authors and do not necessarily represent those of their affiliated organizations, or those of the publisher, the editors and the reviewers. Any product that may be evaluated in this article, or claim that may be made by its manufacturer, is not guaranteed or endorsed by the publisher.
