# Supplementary material for: Baicalin Induces a Potent Innate Immune Response to Inhibit Respiratory Syncytial Virus Replication via Regulating Viral Non-Structural 1 and Matrix RNA
Source: Front Immunol. 2022 Jun 23;13:907047. doi: 10.3389/fimmu.2022.907047 (PMC9259847; doi:10.3389/fimmu.2022.907047)
Supplement: Supplementary file 1 [file DataSheet_1.docx]

**
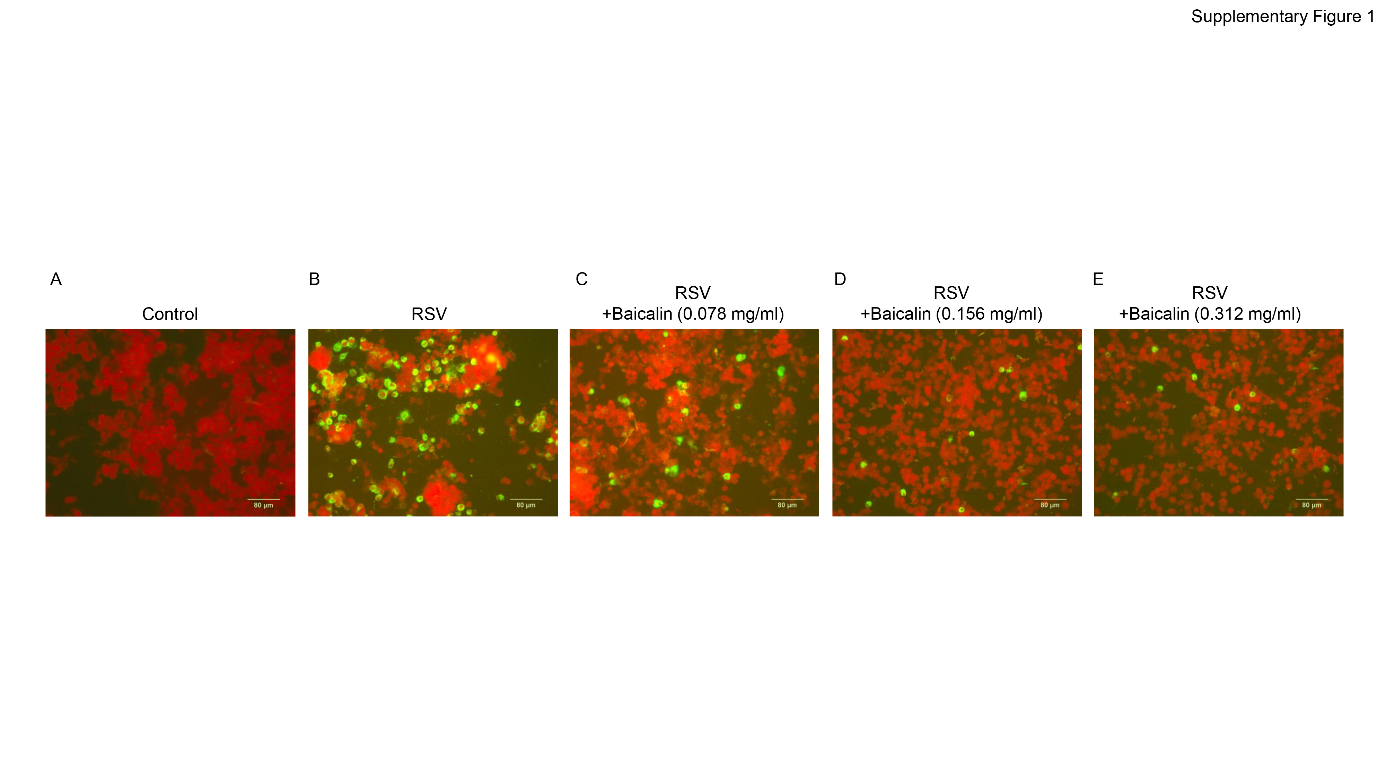
**

**Supplementary Figure 1.** Baicalin effectively inhibited *in vitro* RSV infection by attenuating viral replication. HEp-2 cells were uninfected or infected with RSV (MOI = 3) and then processed with indicated doses of baicalin for 72 h. Cells were then fixed and treated for the analysis of fluorescence microscopy by employing an anti-RSV N protein antibody. Scale bar = 80 µm.

**
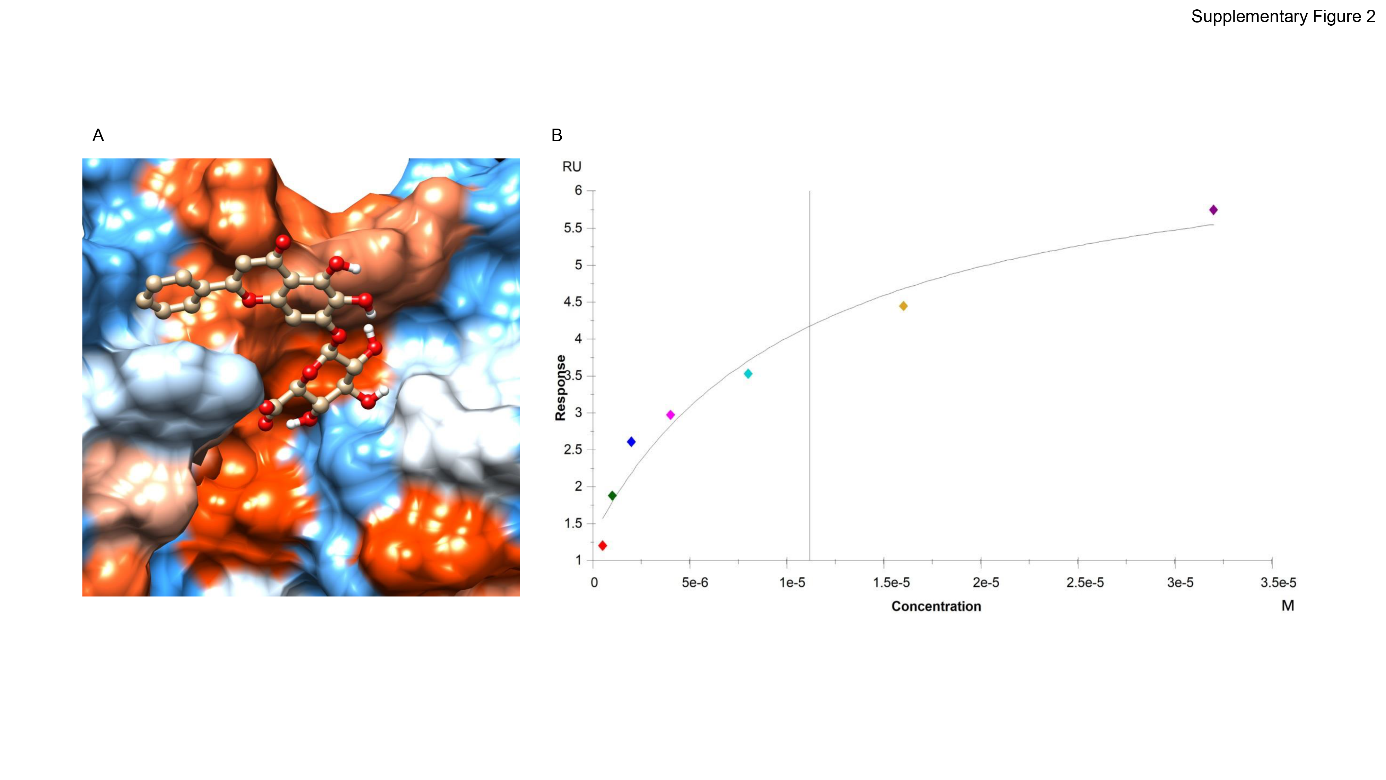
**

**Supplementary Figure 2.** (**A**) Hydrophobic matching diagram of baicalin and NS1 protein. Blue indicates hydrophilicity, and orange indicates hydrophobicity. (**B**) Fitting curve of protein NS1 and baicalin. KD is the affinity constant, calculated from the ratio of kd/ka and was 1.119 × 10^−5^ M. Of note, KD and affinity are inversely related: the smaller the KD value, the greater the affinity.
