# Supplementary material for: Baicalin Induces a Potent Innate Immune Response to Inhibit Respiratory Syncytial Virus Replication via Regulating Viral Non-Structural 1 and Matrix RNA
Source: Front Immunol. 2022 Jun 23;13:907047. doi: 10.3389/fimmu.2022.907047 (PMC9259847; doi:10.3389/fimmu.2022.907047)
Supplement: Supplementary file 2 [file DataSheet_2.docx]

RAW DATA links:

https://www.jianguoyun.com/p/DZAczYcQyIq1ChjuvrQE
